# Influence of maternal folate depletion on Art3 DNA methylation in the murine adult brain; potential consequences for brain and neurocognitive health

**DOI:** 10.1093/mutage/geae007

**Published:** 2024-02-28

**Authors:** Dieuwertje E Kok, Rachael Saunders, Andrew Nelson, Darren Smith, Dianne Ford, John C Mathers, Jill A McKay

**Affiliations:** Division of Human Nutrition and Health, Wageningen University & Research, P.O. Box 17, 6700 AA Wageningen Stippeneng 4, 6708 WE Wageningen Wageningen Campus l Building 124 (Helix), Wageningen, The Netherlands; Department of Applied Sciences, Faculty of Health and Life Sciences, Northumbria University, Northumberland Building, Newcastle Upon Tyne, NE1 8ST, United Kingdom; Department of Applied Sciences, Faculty of Health and Life Sciences, Northumbria University, Northumberland Building, Newcastle Upon Tyne, NE1 8ST, United Kingdom; Department of Applied Sciences, Faculty of Health and Life Sciences, Northumbria University, Northumberland Building, Newcastle Upon Tyne, NE1 8ST, United Kingdom; Department of Applied Sciences, Faculty of Health and Life Sciences, Northumbria University, Northumberland Building, Newcastle Upon Tyne, NE1 8ST, United Kingdom; Human Nutrition & Exercise Research Centre, Centre for Healthier Lives, Population Health Sciences Institute, Newcastle University, Room M2.060, 2nd floor William Leech Building, Framlington Place, Newcastle upon Tyne, NE2 4HH, United Kingdom; Department of Applied Sciences, Faculty of Health and Life Sciences, Northumbria University, Northumberland Building, Newcastle Upon Tyne, NE1 8ST, United Kingdom

**Keywords:** brain, maternal, folate, DNA methylation, developmental origins, neurocognition

## Abstract

The developmental origins of health and disease hypothesis suggest early-life environment impacts health outcomes throughout the life course. In particular, epigenetic marks, including DNA methylation, are thought to be key mechanisms through which environmental exposures programme later-life health. Adequate maternal folate status before and during pregnancy is essential in the protection against neural tube defects, but data are emerging that suggest early-life folate exposures may also influence neurocognitive outcomes in childhood and, potentially, thereafter. Since folate is key to the supply of methyl donors for DNA methylation, we hypothesize that DNA methylation may be a mediating mechanism through which maternal folate influences neurocognitive outcomes. Using bisulphite sequencing, we measured DNA methylation of five genes (*Art3, Rsp16, Tspo, Wnt16,* and *Pcdhb6*) in the brain tissue of adult offspring of dams who were depleted of folate (*n* = 5, 0.4 mg folic acid/kg diet) during pregnancy (~19–21 days) and lactation (mean 22 days) compared with controls (*n* = 6, 2 mg folic acid/kg diet). Genes were selected as methylation of their promoters had previously been found to be altered by maternal folate intake in mice and humans across the life course, and because they have potential associations with neurocognitive outcomes. Maternal folate depletion was significantly associated with *Art3* gene hypomethylation in subcortical brain tissue of adult mice at 28 weeks of age (mean decrease 6.2%, *P* = .03). For the other genes, no statistically significant differences were found between folate depleted and control groups. Given its association with neurocognitive outcomes, we suggest *Art3* warrants further study in the context of lifecourse brain health. We have uncovered a potential biomarker that, once validated in accessible biospecimens and human context, may be useful to track the impact of early-life folate exposure on later-life neurocognitive health, and potentially be used to develop and monitor the effects of interventions.

## Introduction

There has been much support for the developmental origins of health and disease (DOHaD) hypothesis, through which it is postulated that early-life exposures impact health outcomes throughout the life course [1, 2]. Within this paradigm, it is suggested that pre- and peri-natal factors alter early programming of the offspring which is manifest as altered health in later life, although it is still not fully clear through what mechanisms this is achieved.

One set of key mechanisms suggested to play a role in early-life programming are epigenetic modifications [2]. These genome modifications do not involve changes to the DNA sequence but are able to influence gene expression. DNA methylation, the most well-studied of the epigenetic marks, is an important gene regulatory mechanism through which methylation of CpG-rich regions can lead to gene silencing via the inhibition of binding of the regulatory machinery for transcription [3]. It has been well established that these DNA methylation marks are readily influenced by environmental factors, and as such can be considered the interface between the social and physical environment, the genome, and health outcomes [4, 5]. Furthermore, as they are established during foetal and early life, this is considered a critical period through which environmental cues may program these regulatory marks [3]. Indeed, some animal studies have concomitantly reported altered epigenetic, gene expression, and phenotypic outcome measures in response to early-life environmental cues. For example, Nguyen et al. reported global DNA hypermethylation and altered expression of some genes involved in epigenetic processes in the whole brain and hippocampus in offspring whose mothers were exposed to tobacco smoke continuously during pregnancy, whilst also observing neurological deficits in offspring [6]. Additionally, a study investigating the influence of maternal alcohol consumption during pregnancy in mice reported altered expression of 23 genes at 28 days of age together with altered methylation of some of these selected as candidates for further investigation in the hippocampus [7]. The same study also observed that the left hippocampal volume was increased whilst the left olfactory bulb was decreased at 60 days of age in response to maternal ethanol exposure [7]. While some early-life environmentally regulated epigenetic changes may be transient, and likely to influence long-term health through structural and physiological changes, other persistent changes may be latent, with their impact revealed only at a later point in the life course [3]. The long-term impact of such persistent epigenetic changes may be triggered by intrinsic or external factors such as the biological changes associated with ageing or due to the gradual biological impact of long-term exposures to environmental factors such as smoking or diet [8, 9].

Folate, a B vitamin found in green leafy vegetables, is essential for healthy foetal development, with deficiency during pregnancy being associated with an increased risk of neural tube defects [10], isolated orofacial clefts, and neurodevelopmental disorders [11]. Other health or disease outcomes have been associated with maternal folate intake or status during pregnancy, including child BMI [12], asthma [13, 14], and childhood cancers, including leukaemia [15–18], neuroblastoma [19–23] and brain tumours[24–27]. In addition, several childhood outcomes associated with brain function and development have been associated with maternal folate intake or status during pregnancy including cognition [28, 29], motor skills [30], language and communication [28, 31–33], resilience and emotional intelligence [34], neurodevelopment and autism [35–37]. As several early-life neurocognitive parameters including cognitive and academic performance [38–41] are predictive for later life neurocognitive disorders such as Alzheimer’s disease [42], it is highly plausible that maternal folate intake or status during pregnancy can have a life-long influence on brain health. However, while there is evidence to suggest that higher folate status in adulthood is protective against adverse neurocognitive outcomes in later life [43–45], it is not yet known if early life, particularly maternal, folate intake or status influences cognitive outcomes in later years.

Folate is a key player in one-carbon metabolism, that is required for the production of the universal methyl donor, S-adenosylmethionine, the substrate required for the methylation of DNA [46]. Therefore, folate availability influences the establishment and maintenance of DNA methylation patterns. Indeed, maternal folate levels during pregnancy have been associated with variation in offspring DNA methylation in the offspring in both short and long term [47–51]. More specifically, methylation of genes involved with neurodevelopment has been associated with folate intake during pregnancy in rats and humans [52, 53], as well as *in vitro* [54], suggesting a key mechanism through which folate may influence brain health.

Previously we uncovered five gene promoters (*Art3, Rsp16, Tspo, Wnt16*, and *Pcdhb6*) which were modestly hypermethylated in response to low maternal folate during early development and in adulthood across both mouse (liver) and human studies (cord blood and/or saliva) [55]. The function of most of these genes implies that they are involved in neurocognition (see Table 1 for details), and therefore may represent persistent, latent epigenetic changes that have the potential to influence neurocognitive outcomes in later life. Here we have used bisulphite sequencing to measure DNA methylation in the promoter regions of these five genes in the brain tissue of murine adult offspring in response to maternal folate depletion during pregnancy. Previously, in adult offspring from this mouse study we have observed lower plasma glucose, increased plasma triacylglycerol [69], changes to hepatic DNA methylation, gene expression profiles [50], and slightly increased oxidative DNA damage levels in subcortical brain regions [70] in response to maternal folate depletion during pregnancy and lactation.

**Table 1.** Description of genes studied.

Gene symbol	Gene name	Protein function	Evidence for a role in neurological conditions, neurocognition and other brain-related parameters
*Art3*	ADP-ribosyltransferase 3	Adds/removes ADP-ribose to/from an arginine residue to regulate protein function	Hypomethylated in established schizophrenia [56] and maltreated children [57] compared to controls. SNPs within this gene have been associated with educational attainment [58] and hippocampal volume [59].
*Pcdhb6*	Protocadherin beta 6	Neural cadherin-like cell adhesion protein	Likely to play a role in the establishment and function of specific cell–cell neural connections. Disruption of cell adhesion pathways involving *Pcdhb6* occur in Ms5Yah mice which exhibit motor coordination deficits, and spatial learning and memory impairments [60].
*Rps16*	Ribosomal protein S16	Component of the 40S ribosomal subunit, therefore plays a role in protein synthesis.	No current evidence found.
*Tspo*	Translocator protein	Facilitates the movement of cholesterol into mitochondria to permit the initiation of steroid hormone synthesis	Associated disorders include hepatic encephalopathy and generalized anxiety disorder. The TSPO protein may be a predictive marker of amyloid pathology linked to Alzheimer’s disease [61].
*Wnt16*	Wingless-type MMTV integration site family, member 16	Member of the Wnt signalling protein family that are implicated in oncogenesis and in developmental processes, including regulation of cell fate during embryogenesis.	Hypermethylated in maltreated children compared to controls [57]. SNPs within this gene have been associated with brain volume, functional and neuroimaging measurements [62–68].

## Materials and methods

### Experimental diets and mouse tissue collection

Newcastle University Ethics Review Committee and the UK Home Office (Project licence number 60/3979) approved all animal procedures which have been described previously [69, 70]. The animals were housed in the Comparative Biology Centre, Newcastle University with 12 h light and dark cycles at 20–22°C and fresh water available ad libitum. Adult (i.e. over 8 weeks old) female C57BL/6J mice were allocated at random to either a normal folate diet (2 mg folic acid/kg diet, *n* = 23) or low folate (0.4 mg folic acid/kg diet, *n* = 16) (6 g/d/mouse) and maintained on this diet for 4 weeks prior to mating, during pregnancy, and lactation until weaning (mean 22 days post-partum). Post-weaning all animals included in the current study were fed diets containing adequate amounts of folate (control diets with 2 mg folic acid/kg diet). Diet compositions were modified from AIN-93G24 and have been described previously [49]. All ingredients, other than folic acid, were included in both diets at the same concentrations to avoid potential confounding.

Sample collection and confirmation of folate depletion [69] have been detailed previously. Briefly, adult offspring were killed at 28 weeks of age for tissue collection. The brain was removed, weighed, and dissected (hippocampus, cortex, cerebellum, and subcortical regions) and snap frozen in liquid nitrogen and stored at −80°C prior to DNA extraction.

### Extraction, bisulphite modification of DNA

DNA was extracted from ground subcortical brain tissue (as other brain tissue sections had been utilized for studies elsewhere) from 11 adult murine offspring (five folate depleted during pregnancy and lactation (four females, one male) and six controls (all female). Animals were selected based on the remaining archived tissue available, although it would be preferable to use samples from both male and female mice in each group this was not possible). Extraction was performed using the E.Z.N.A.® Tissue DNA Kit (Omega Biotech) in accordance with the manufacturer’s instructions. A total of 2 µg DNA was bisulphite modified using the EZ DNA Methylation Gold kit (Zymo Research) following the manufacturer’s instructions, with a minor modification for the elution step (using 12 µl rather than 10 µl). Prior to use in PCR reactions, bisulphite modified DNA was diluted 1:20 (4 µl in 76 µl water) to give an approximate concentration of 8 ng/µl.

### PCR

Bisulfite sequencing was used to determine the percentage methylation at individual CpG sites within the *Art3*, *Pcdhb6*, *Rsp16*, *Tspo*, and *Wnt16* promoters. Details of PCR conditions and primers are detailed in Table 2. Briefly, 1 µl of diluted bisulphite-treated DNA was added as a template in a PCR reaction using 10 µl Hot Star Taq mastermix (Qiagen), 1 µl of forward, and reverse primers (at a concentration of 10 pmol/µl) in a total volume of 20 µl. Amplification was carried out in a Mastercycler Nexus Gradient thermocycler (Eppendorf) using the following protocol; 95°C for 15 min, then 40 cycles of 95°C for 30 s, annealing temperature for 1 min, 72°C for 1 min, followed by 72°C for 5 min. Three microlitres of PCR product was used for visualization on a 1% agarose-TAE gel subjected to electrophoresis to confirm the presence of a PCR product. The remaining 17 µl PCR products were subsequently purified for sequencing using Thermo Scientific GeneJET™ PCR Purification Kit (Fisher Scientific, UK) to remove primers, dNTPs, and other impurities from the PCR product, according to the manufacturers’ instructions.

**Table 2.** PCR conditions,

Gene	Forward primer (5ʹ-3ʹ)	Reverse primer (5ʹ-3ʹ)	Size (bp)	Annealing temperature (°C)
*Art3*	GGAGTTAGAATTTGGGGAAGAGTAT	CCCTCACTCCTACTAAAACCAATC	229	60
*Pcdhb6*	ATTTTTGGGATTTAGAATGTTGAAT	AAAAAACCACTTACCTTTTCTCTAAC	230	55
*Rsp16*	AGTTTATGGGTGTGAGGTAGTTTTT	ACCAAAACCTCCAAACTTTTTAAAT	316	55
*Tspo*	TGGGTAGAATTGAAATTTTTAGTGG	CATACCTAACTTCCACAACCCTAAC	262	55
*Wnt16*	GTTTTTTTTATAAGGAAGAAATGTTTTTAT	CAAAATACACCCATAACTATTCCAC	164	55

### Sequencing

Library preparation and sequencing were carried out at NU-OMICS DNA Sequencing research facility (Northumbria University). Purified PCR products were quantified using Quant-iT™ PicoGreen™ dsDNA Assay (Invitrogen) and normalized to 20 nM and pooled by experimental condition. Libraries of pooled amplicons were prepared using the NEBNext® Ultra™ II DNA Library Prep Kit for Illumina (New England Biolabs). The quality of prepared libraries was assessed using the Agilent High Sensitivity DNA Kit Guide (Agilent) and quantified using Quant-iT™ PicoGreen™ dsDNA Assay (Invitrogen). Libraries were normalized to 4 nM, pooled, and sequenced using the Illumina MiSeq V2 300 cycle chemistry. Fastq files containing sequencing data were uploaded to EPIC TABSTAT v1.7 (https://tabsat.ait.ac.at/) for analysis in paired-end mode with mouse mm10 genome used as the reference and Bowtie2 as the mapping tool. The Bismark output files produced % methylation at each CpG site sequenced for each sample.

### Statistical analysis

Power calculations to determine the required number of animals per group were carried out based on the data for cg14855841 (ART3) from the AFAST study, in which the standard deviation was calculated as 0.68. Considering a minimum effect size = 1.6% methylation between groups, 80% power, alpha = 0.05, the minimum sample/group = 5. All statistical analyses were performed using IBM SPSS Statistics 26 and a *P*-value of < .05 was considered statistically significant. One-sample Kolmogorov–Smirnov tests were applied to CpG-specific and mean methylation data to determine the distribution. For each gene, either or both Pearson’s (normal data) or Spearman (non-normal data) correlations were used to assess correlation the between percentage methylation at individual CpGs and mean percentage methylation, depending on the distribution of the data. To reduce the possibility for false positive associations through multiple testing, where mean methylation was highly correlated with that of individual CpGs across a gene region, initially mean methylation across a given gene was analysed for mice exposed to low folate *in utero* and during weaning and those exposed to a normal folate diet. For any genes where statistically significant differences between diet groups for mean methylation were observed, or where methylation at individual CpG sites did not correlate with mean methylation, data for each individual CpG were analysed separately. Where data were found to be normally distributed, comparisons of percentage methylation were analysed using univariate ANOVA. Where data were not normally distributed, comparisons of percentage methylation were analysis using independent samples Mann–Whitney *U* test.

## Results

### Influence of maternal folate depletion on gene-specific methylation in the adult brain

**Figure 1.**
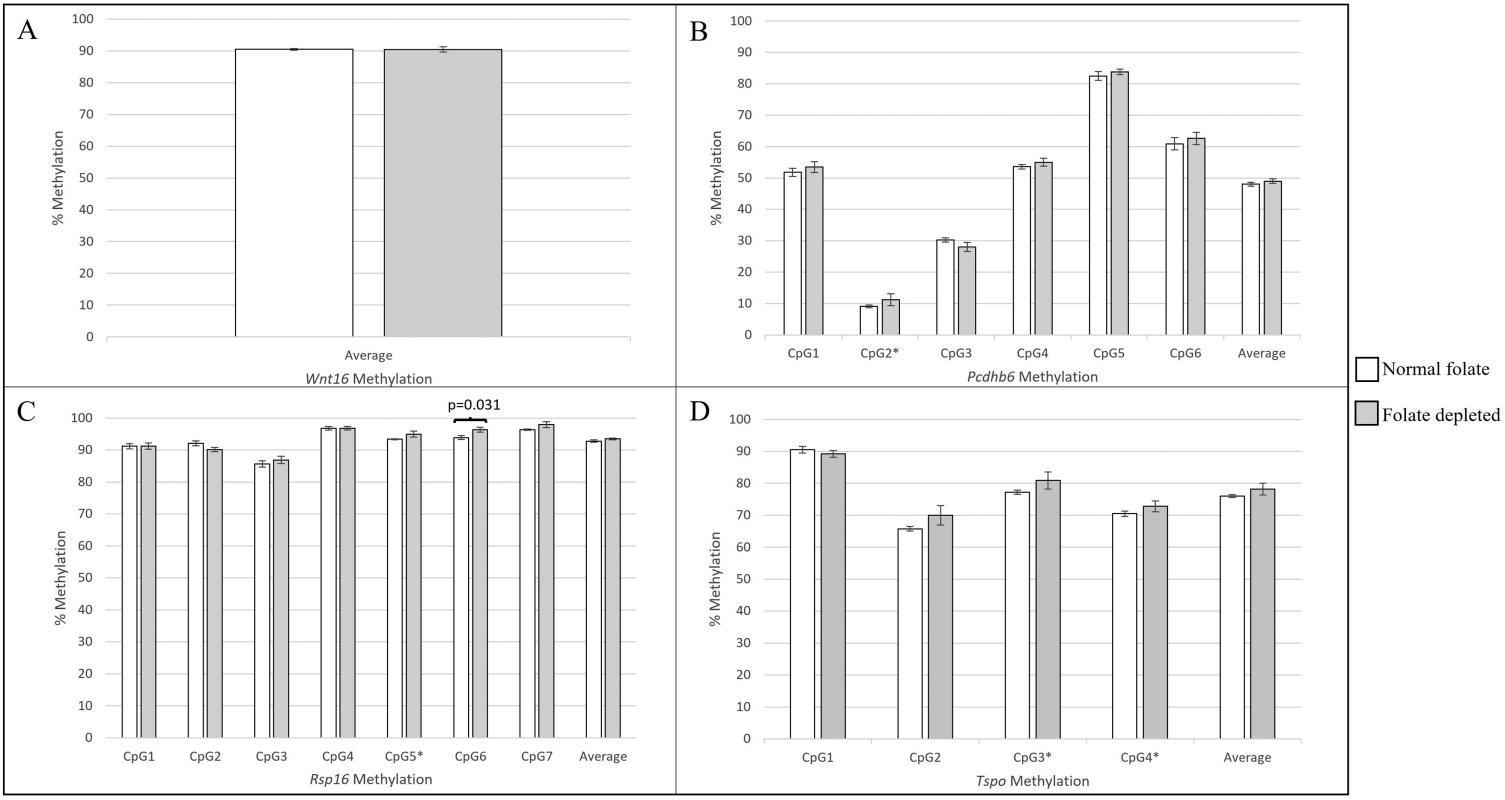
Effect of maternal folate depletion during pregnancy and lactation on A) *Wnt16*, B) *Pcdhb6*, C) *Rsp16* and D) *Tspo* methylation in brain tissue of adult offspring. Number of mice: *n* = 6 normal folate (white bars), *n* = 5 folate depletion (grey bars). Univariate ANOVA were used to assess differences in methylation at individual CpGs and across all CpGs in the region assessed (i.e. mean), with the except of CpGs for which data were not normally distributed (indicated by * in the figure), in that case Mann–Whitney *U* tests were used.

Results of one-sample Kolmogorov–Smirnov tests for data distribution and correlation between methylation sites within the same gene are shown in, Supplementary Table S1, File 1, and Supplementary Tables S2 and S3, File 2, respectively. As all CpGs measured in *Wnt16* were highly correlated with mean methylation across the locus in the adult brain, mean methylation was assessed in response to maternal folate depletion. Compared with mice born to dams fed the normal folate diet, there was no difference in mean *Wnt16* methylation in those born to folate depleted dams; mean methylation was 90.41% (SEM = 0.754) and 90.46% (SEM = 0.349) for normal and folate depleted, respectively (*P* = .954, Fig. 1A).

As only one CpG correlated with mean methylation for both *Pcdhb6* and *Rsp16,* each CpG was assessed individually, and as a mean across all CpG sites within each gene. Folate depletion did not significantly influence methylation at most individual CpGs or across mean methylation for these genes (Fig. 1B and C), with only CpG6 of the *Rsp16* locus being statistically significantly hypermethylated in response to folate depletion compared with normal folate (96.39% (SEM = 0.787) vs 93.88% (SEM = 0.612), respectively, *P* = .031).

For the *Tspo* gene, methylation of all CpGs analysed, except CpG 1, correlated with mean methylation across CpG sites. Consequently, we investigated mean methylation and methylation of CpG 1 for this gene in response to maternal folate depletion. Neither methylation at CpG 1 specifically nor mean methylation across the gene region differed in response to maternal folate depletion (CpG1 90.49% (SEM = 0.914) vs 89.21% (SEM = 1.140), *P* = .400; mean methylation 75.98% (SEM = 1.865) vs 78.22% (SEM = 0.491), *P* = 0.266 for normal vs folate depleted groups, respectively, Fig. 1D).

**Figure 2.**
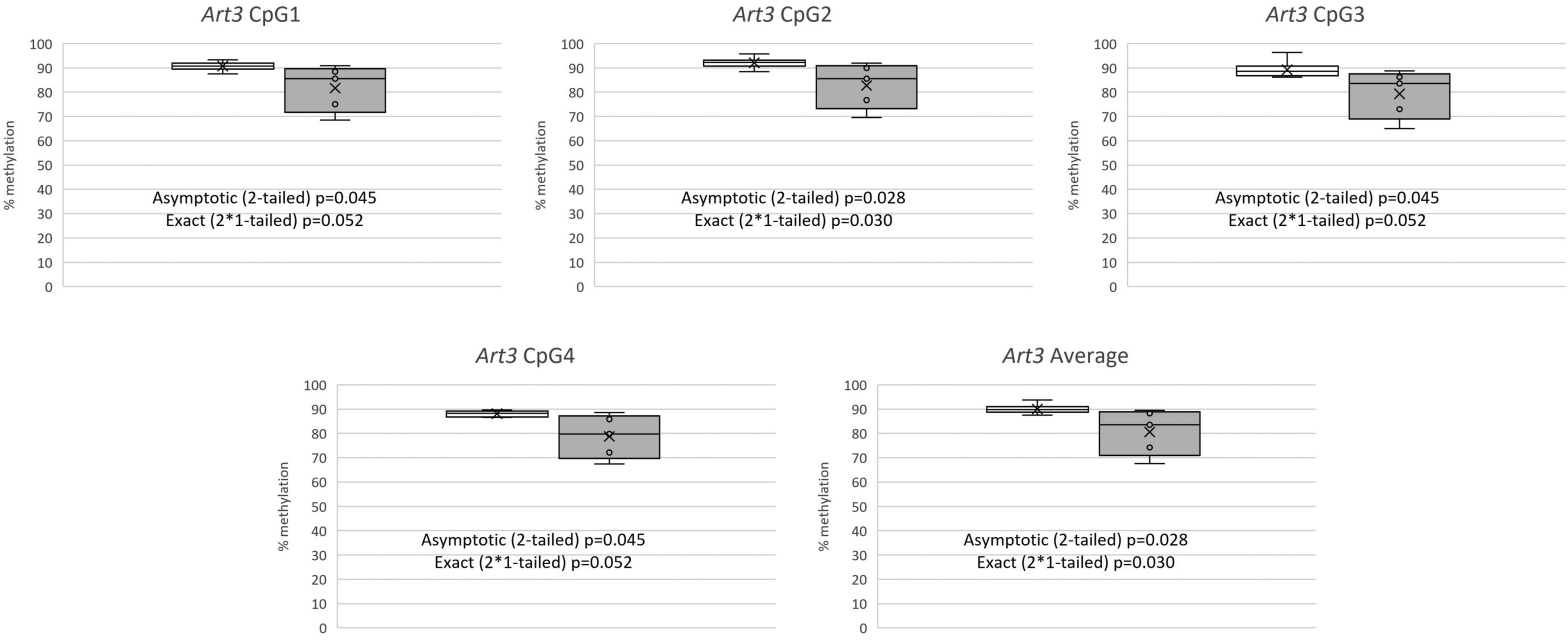
Box and whisker plots to illustrate the effect of maternal folate depletion during pregnancy and lactation on *Art3* methylation at specific CpG sites, and the mean for all measured CpGs in brain tissue of adult offspring. Number of mice: *n* = 6 normal folate (white box and whisker), *n* = 5 folate depletion (grey box and whisker). Mann–Whitney *U* tests were used to assess differences in methylation at individual CpGs and across all CpGs in the region assessed (i.e. average).

As all CpGs were highly correlated with mean methylation across the *Art3* locus in the adult brain, mean methylation was assessed in response to maternal folate depletion. Mean methylation across the *Art3* region measured was statistically significantly lower (hypomethylated) in response to maternal folate depletion compared with offspring born to normal folate-fed dams (Fig. 2, median 83.59% and 89.76%, respectively, asymptotic *P* = .028 or exact *P* = .030 assessed via Mann–Whitney *U* test). CpGs in *Art3* were also assessed individually. All individual CpGs were hypomethylated in response to maternal folate depletion, with CpG 3 showing a statistically significant difference (Fig. 2, asymptotic *P* = .028 or exact *P* = .030 assessed), while CpGs 1, 2, and 4 were all on the borderline of significance (Fig. 2, asymptotic *P* = .045 or exact *P* = .052).

## Discussion

Here we quantified DNA methylation of five target genes in the sub-cortical brain tissue of adult mice whose mothers had been depleted of folate during pregnancy and lactation. These target genes were selected as they have previously been found to have altered methylation in other tissues in association with early-life folate exposure in both mice and humans, and, for 4/5 genes, also in adulthood [47, 48, 50, 51, 55]. Moreover, our previous investigation suggested these genes may potentially influence neurocognitive outcomes [55]. Given the evidence that early-life folate status may influence neurocognitive outcomes in offspring [28–37], we hypothesized that altered DNA methylation of these genes within brain tissue may mediate this relationship.

While previously we reported increased methylation in both fetal and adult liver in response to low maternal folate across all genes assessed, in the brain we observed statistically significant hypermethylation at a single CpG within the *Rsp16* gene promoter, and mean hypomethylation across all four CpGs measured within the *Art3* gene promoter in response to maternal folate depletion, which was statistically significant also at one of these CpGs and of borderline significance at the other three. Given the variability in methylation in response to low maternal folate across the *Rsp16* gene (Fig. 1), together with the lack of significant effects at other CpGs across the gene region, it is possible that this finding is due to chance. However, for *Art3*, hypomethylation across the locus was consistent at all CpGs investigated, which makes it unlikely that this finding is due to chance. In addition to our previous report of *Art3* hypermethylation in the liver of offspring born to folate-depleted mothers, hypermethylation of the promoter of this gene was observed in cord blood and saliva of the offspring of mothers with lower folate status [55]. Although here we have observed an opposing direction of methylation change in brain tissue, taken together, these data suggest that methylation of *Art3* is particularly susceptible to maternal folate status in multiple tissues and across species. This is not the first observation of a tissue-specific change in methylation in response to folate exposure. Previously, we reported a significant interaction between tissue type and dietary folate on methylation of the *Igf2* differentially methylated region 2 (DMR2) in folate-depleted female mice, where we observed hypomethylation in blood, hypermethylation in liver, and no methylation change in kidney tissue in response to folate depletion [71]. Moreover, the complexities of methylation differences across tissues in response to maternal exposures, including folate, has been documented at the *Igf2/H19* locus in human offspring, whereby directionality of methylation change was found to vary across loci and tissues and was specific to a given exposure [72]. Our findings therefore reinforce the complexities of exposure-DNA methylation relationships, emphasising the importance of investigating relevant tissues. The changes that we have observed in *Art3* methylation in the brain here, may have implications for neurocognitive outcomes.

Although the function of ART3 protein as an ADP ribosyltransferase, catalysing the addition or removal of ADP-ribose to arginine residues of proteins, may not initially point to a clear role in neurocognition, its utilization of NAD for catalysis links it to NAD metabolism [73]. NAD metabolism has recently emerged as a potential target for age-associated diseases, including cognitive decline, because lower NAD status has been associated with adverse neurocognitive outcomes (reviewed in [74]). This is unsurprising because NAD is essential in providing energy for multiple cellular functions, including metabolic pathways, DNA repair, chromatin remodelling, cellular senescence, and immune cell function that can decline during ageing [74]. Although speculative, it is possible that *Art3* hypomethylation may lead to increased Art3 expression and therefore lower NAD status, which warrants further investigation. Therefore, it is plausible that ART3 may functionally influence neurocognition through its role in NAD metabolism.

In line with this suggestion, the *ART3* gene has been associated with several outcomes related to neurocognition. In a recent large meta-analysis, methylation at cg22301128 located within the gene body of *ART3* was reported to be significantly hypomethylated in blood samples from cases with established schizophrenia compared to controls [56]. Also, DNA methylation at cg12159836 within the 5ʹUTR of *ART3* was also observed to be significantly lower in saliva from maltreated children, who go on to have increased risk of psychiatric disorders, compared with controls [57]. Additionally, in a study of over three million individuals the rs4859423 T>C SNP within the *ART3* gene was associated with educational attainment, again suggesting that this locus may influence cognitive outcomes [58]. Moreover, in a genome-wide association study investigating endophenotypes associated with pre-diagnostic stages of Alzheimer’s disease, the rs79955867 C > T single nucleotide polymorphism (SNP) mapping to a CpG site within the *ART3* locus was associated with decreased hippocampal volume [59]. As the hippocampus is important for learning and memory, hippocampal volume has been associated with processing speed, working memory, spatial navigation, and abstract reasoning [75–79]. While not directly related to neurocognition, the ART3 protein, a known tumour marker, has been observed in the cerebrospinal fluid of recurrent medulloblastoma patients [80], and expression of this gene has also been associated with survival after diagnosis with medulloblastoma [81]. Thus, in addition to its potential significance for neurocognition, altered methylation of this locus via nutritional modulation may also impact other aspects of brain health.

Here we have extended previous findings from studies of mouse liver and human blood and saliva samples in which we showed that *ART3* methylation was influenced by maternal folate status across the lifecourse, to adult murine brain tissue. Our findings that folate status influences methylation at the *ART3* locus in multiple tissues and in both mice and humans, suggest that this locus may be of value as a biomarker for the impact of early-life exposure to folate on neurocognitive outcomes or, more broadly, brain health. It will be important to establish whether the changes in *Art3* methylation in mouse brain also occur in human brain. This could be achieved using tissues from human brain tissue banks and either linkage to direct measurements of folate status in the donors or using Mendelian randomization approaches as a proxy for folate status [82]. For future epidemiological and intervention studies directly in humans, the assessment of *Art3* methylation in blood, as a proxy for brain methylation, is likely to be the most practical measure. However, given that the direction of methylation change in response to folate appears to differ between tissues, in future studies it will be important to determine whether there are similar changes in *Art3* methylation in the blood and brain in response to folate exposures.

Our use of samples from a well-designed folate intervention study in mice provide confidence that our findings of effects of folate supply during pregnancy and lactation on *Art3* methylation in the brain of adult offspring are likely to be causal. However, our study also has limitations. Although the number of animals (*n* = 5/6) in which brain methylation was measured was within the usual range assessed for the detection of methylation change in rodent studies, and despite power calculations indicating sufficient power for *Art3*, it may have restricted statistical power to be able to detect more subtle changes in the other genes where methylation was measured. While the utilization of archived murine tissue was a particular strength of this study in line with the 3Rs ethos of ethical use of animals in research, we were unable to assess a range of specific brain sections due to the availability of tissues from our archived sample collection, with only material for sub-cortical regions available for analysis here as other sections had been utilized for previous studies. Pinpointing specific regions of the brain in which altered methylation in response to maternal folate occurs is likely to be useful to illuminate the potential impact of this exposure on offspring neurocognition. Moreover, due to the prolonged storage of the archived tissue, we were unable to measure gene expression and investigate relationships with the methylation changes reported here. It will be important to undertake such gene expression studies in the future to understand the potential functional impact of these methylation changes invoked by inadequate maternal folate intake. Another limitation of the use of archived samples was that no neurocognitive data are available to determine if there is an association between the methylation change in response to maternal folate and cognition-related outcomes.

In summary, this study has identified *Art3* as a gene of interest through which maternal folate may influence neurocognition throughout the life course. Such relationships may be mediated by DNA methylation. We hope that these findings will stimulate other researchers to investigate the relationship between folate status and methylation of *Art3*, and more broadly, brain DNA methylation and neurocognitive outcomes. For example, in human studies with data on maternal folate status (using direct measurements or Mendelian Randomisation approaches) and both genome-wide methylation data and neurocognitive data, would provide an invaluable resource for investigating the relationships among maternal folate status, *ART3* methylation and neurocognitive outcomes. By utilizing archived murine samples, we have uncovered a potential biomarker that may be useful to track early-life exposure to folate to later life neurocognitive health, and that may have potential for developing and monitoring nutritional interventions to reduce risk of adverse neurocognitive outcomes in later life. Further studies are therefore warranted to aid understanding of the potential role of the *ART3* gene, including the role of methylation at this locus, in relation to neurocognition across the life course.

## Supplementary data

Supplementary Table X is available at *Mutagenesis* Online.

geae007_suppl_Supplementary_Tables_1

geae007_suppl_Supplementary_Tables_2-3

## Data Availability

Data are available in the manuscript and supplementary material. Raw data are available upon request from the corresponding author.
