# Supplementary material for: Influence of maternal folate depletion on Art3 DNA methylation in the murine adult brain; potential consequences for brain and neurocognitive health
Source: Mutagenesis. 2024 Feb 28;39(3):196–204. doi: 10.1093/mutage/geae007 (PMC11040152; doi:10.1093/mutage/geae007)
Supplement: geae007_suppl_Supplementary_Tables_2-3 [file geae007_suppl_supplementary_tables_2-3.docx]

**Supplementary Table 2.** Pearson’s Correlations between individual CpG’s and average methylation across a given gene region.

| ***WNT16*** | | | | | | | | | |
| --- | --- | --- | --- | --- | --- | --- | --- | --- | --- |
|  | | CpG1 | CpG2 | CpG3 | CpG4 | Average |  | | |
| CpG1 | Pearson Correlation | 1 | **.650^*^** | **.690^*^** | 0.558 | **.921^**^** |  |  |  |
|  | Sig. (2-tailed) |  | **0.030** | **0.019** | 0.075 | **0.000** |  |  |  |
| CpG2 | Pearson Correlation | **.650^*^** | 1 | 0.344 | 0.131 | **.656^*^** |  |  |  |
|  | Sig. (2-tailed) | **0.030** |  | 0.300 | 0.702 | **0.028** |  |  |  |
| CpG3 | Pearson Correlation | **.690^*^** | 0.344 | 1 | 0.483 | **.830^**^** |  |  |  |
|  | Sig. (2-tailed) | **0.019** | 0.300 |  | 0.132 | **0.002** |  |  |  |
| CpG4 | Pearson Correlation | 0.558 | 0.131 | 0.483 | 1 | **.706^*^** |  |  |  |
|  | Sig. (2-tailed) | 0.075 | 0.702 | 0.132 |  | **0.015** |  |  |  |
| Average | Pearson Correlation | **.921^**^** | **.656^*^** | **.830^**^** | **.706^*^** | 1 |  |  |  |
|  | Sig. (2-tailed) | **0.000** | **0.028** | **0.002** | **0.015** |  |  |  |  |
| ***PCDHB6*** | | | | | | | | | |
|  | | CpG1 | CpG2 | CpG3 | CpG4 | CpG5 | CpG6 | Average |  |
| CpG1 | Pearson Correlation | 1 | 0.487 | 0.046 | -0.223 | 0.128 | -0.356 | 0.330 |  |
|  | Sig. (2-tailed) |  | 0.129 | 0.893 | 0.510 | 0.708 | 0.283 | 0.322 |  |
| CpG2 | Pearson Correlation | 0.487 | 1 | -0.032 | 0.050 | -0.122 | 0.193 | 0.529 |  |
|  | Sig. (2-tailed) | 0.129 |  | 0.926 | 0.884 | 0.721 | 0.569 | 0.095 |  |
| CpG3 | Pearson Correlation | 0.046 | -0.032 | 1 | 0.429 | -0.241 | 0.099 | 0.406 |  |
|  | Sig. (2-tailed) | 0.893 | 0.926 |  | 0.188 | 0.476 | 0.772 | 0.215 |  |
| CpG4 | Pearson Correlation | -0.223 | 0.050 | 0.429 | 1 | 0.102 | 0.509 | 0.580 |  |
|  | Sig. (2-tailed) | 0.510 | 0.884 | 0.188 |  | 0.766 | 0.110 | 0.061 |  |
| CpG5 | Pearson Correlation | 0.128 | -0.122 | -0.241 | 0.102 | 1 | 0.481 | 0.468 |  |
|  | Sig. (2-tailed) | 0.708 | 0.721 | 0.476 | 0.766 |  | 0.134 | 0.147 |  |
| CpG6 | Pearson Correlation | -0.356 | 0.193 | 0.099 | 0.509 | 0.481 | 1 | **.691^*^** |  |
|  | Sig. (2-tailed) | 0.283 | 0.569 | 0.772 | 0.110 | 0.134 |  | **0.019** |  |
| Average | Pearson Correlation | 0.330 | 0.529 | 0.406 | 0.580 | 0.468 | **.691^*^** | 1 |  |
|  | Sig. (2-tailed) | 0.322 | 0.095 | 0.215 | 0.061 | 0.147 | **0.019** |  |  |
| ***RSP16*** | | | | | | | | | |
|  | | CpG1 | CpG2 | CpG3 | CpG4 | CpG5 | CpG6 | CpG7 | Average |
| CpG1 | Pearson Correlation | 1 | 0.179 | 0.309 | -0.132 | 0.135 | -0.250 | -0.008 | 0.466 |
|  | Sig. (2-tailed) |  | 0.599 | 0.356 | 0.698 | 0.692 | 0.458 | 0.981 | 0.148 |
| CpG2 | Pearson Correlation | 0.179 | 1 | -0.039 | 0.162 | -0.343 | -0.099 | -0.024 | 0.282 |
|  | Sig. (2-tailed) | 0.599 |  | 0.910 | 0.633 | 0.302 | 0.771 | 0.944 | 0.401 |
| CpG3 | Pearson Correlation | 0.309 | -0.039 | 1 | -0.547 | -0.131 | 0.122 | 0.121 | 0.459 |
|  | Sig. (2-tailed) | 0.356 | 0.910 |  | 0.082 | 0.701 | 0.720 | 0.723 | 0.155 |
| CpG4 | Pearson Correlation | -0.132 | 0.162 | -0.547 | 1 | 0.237 | 0.372 | 0.011 | 0.187 |
|  | Sig. (2-tailed) | 0.698 | 0.633 | 0.082 |  | 0.483 | 0.260 | 0.975 | 0.583 |
| CpG5 | Pearson Correlation | 0.135 | -0.343 | -0.131 | 0.237 | 1 | 0.585 | 0.224 | 0.483 |
|  | Sig. (2-tailed) | 0.692 | 0.302 | 0.701 | 0.483 |  | 0.059 | 0.507 | 0.132 |
| CpG6 | Pearson Correlation | -0.250 | -0.099 | 0.122 | 0.372 | 0.585 | 1 | 0.255 | **.611^*^** |
|  | Sig. (2-tailed) | 0.458 | 0.771 | 0.720 | 0.260 | 0.059 |  | 0.449 | **0.046** |
| CpG7 | Pearson Correlation | -0.008 | -0.024 | 0.121 | 0.011 | 0.224 | 0.255 | 1 | 0.489 |
|  | Sig. (2-tailed) | 0.981 | 0.944 | 0.723 | 0.975 | 0.507 | 0.449 |  | 0.127 |
| Average | Pearson Correlation | 0.466 | 0.282 | 0.459 | 0.187 | 0.483 | **.611^*^** | 0.489 | 1 |
|  | Sig. (2-tailed) | 0.148 | 0.401 | 0.155 | 0.583 | 0.132 | **0.046** | 0.127 |  |
| ***TSPO*** | | | | | | | | | |
|  | | CpG1 | CpG2 | CpG3 | CpG4 | Average |  | | |
| CpG1 | Pearson Correlation | 1 | .019 | -.152 | -.305 | .061 |  |  |  |
|  | Sig. (2-tailed) |  | .955 | .655 | .362 | .859 |  |  |  |
| CpG2 | Pearson Correlation | .019 | 1 | **.870^**^** | **.858^**^** | **.970^**^** |  |  |  |
|  | Sig. (2-tailed) | .955 |  | **.000** | **.001** | **.000** |  |  |  |
| CpG3 | Pearson Correlation | -.152 | **.870^**^** | 1 | **.891^**^** | **.940^**^** |  |  |  |
|  | Sig. (2-tailed) | .655 | **.000** |  | **.000** | **.000** |  |  |  |
| CpG4 | Pearson Correlation | -.305 | **.858^**^** | **.891^**^** | 1 | **.893^**^** |  |  |  |
|  | Sig. (2-tailed) | .362 | **.001** | **.000** |  | **.000** |  |  |  |
| Average | Pearson Correlation | .061 | **.970^**^** | **.940^**^** | **.893^**^** | 1 |  |  |  |
|  | Sig. (2-tailed) | .859 | **.000** | **.000** | **.000** |  |  |  |  |

*. Correlation is significant at the 0.05 level (2-tailed).

**. Correlation is significant at the 0.01 level (2-tailed).

**Supplementary Table 3.** Spearman’s rho Correlations between individual CpG’s and average methylation across a given gene region.

| ***TSPO*** | | | | | | |  |
| --- | --- | --- | --- | --- | --- | --- | --- |
|  | | CpG1 | CpG2 | CpG3 | CpG4 | Average | |
| CpG1 | Correlation Coefficient | 1.000 | 0.118 | -0.091 | -0.264 | 0.082 | |
|  | Sig. (2-tailed) |  | 0.729 | 0.790 | 0.433 | 0.811 | |
| CpG2 | Correlation Coefficient | 0.118 | 1.000 | **.673^*^** | **.718^*^** | **.964^**^** | |
|  | Sig. (2-tailed) | 0.729 |  | **0.023** | **0.013** | **0.000** | |
| CpG3 | Correlation Coefficient | -0.091 | **.673^*^** | 1.000 | **.618^*^** | **.791^**^** | |
|  | Sig. (2-tailed) | 0.790 | **0.023** |  | **0.043** | **0.004** | |
| CpG4 | Correlation Coefficient | -0.264 | **.718^*^** | **.618^*^** | 1.000 | **.791^**^** | |
|  | Sig. (2-tailed) | 0.433 | **0.013** | **0.043** |  | **0.004** | |
| Average | Correlation Coefficient | 0.082 | **.964^**^** | **.791^**^** | **.791^**^** | 1.000 | |
|  | Sig. (2-tailed) | 0.811 | **0.000** | **0.004** | **0.004** |  | |
| ***ART3*** | | | | | | |  |
|  | | CpG1 | CpG2 | CpG3 | CpG4 | Average | |
| CpG1 | Correlation Coefficient | 1.000 | **.991^**^** | **.818^**^** | **.836^**^** | **.991^**^** | |
|  | Sig. (2-tailed) |  | **0.000** | **0.002** | **0.001** | **0.000** | |
| CpG2 | Correlation Coefficient | **.991^**^** | 1.000 | **.864^**^** | **.800^**^** | **1.000^**^** | |
|  | Sig. (2-tailed) | **0.000** |  | **0.001** | **0.003** |  | |
| CpG3 | Correlation Coefficient | **.818^**^** | **.864^**^** | 1.000 | 0.555 | **.864^**^** | |
|  | Sig. (2-tailed) | **0.002** | **0.001** |  | 0.077 | **0.001** | |
| CpG4 | Correlation Coefficient | **.836^**^** | **.800^**^** | 0.555 | 1.000 | **.800^**^** | |
|  | Sig. (2-tailed) | **0.001** | **0.003** | 0.077 |  | **0.003** | |
| Average | Correlation Coefficient | **.991^**^** | **1.000^**^** | **.864^**^** | **.800^**^** | 1.000 | |
|  | Sig. (2-tailed) | **0.000** |  | **0.001** | **0.003** |  | |

*. Correlation is significant at the 0.05 level (2-tailed).

**. Correlation is significant at the 0.01 level (2-tailed).
